# Fine-Grained Motion Recognition in At-Home Fitness Monitoring with Smartwatch: A Comparative Analysis of Explainable Deep Neural Networks

**DOI:** 10.3390/healthcare11070940

**Published:** 2023-03-24

**Authors:** Seok-Ho Yun, Hyeon-Joo Kim, Jeh-Kwang Ryu, Seung-Chan Kim

**Affiliations:** 1 Department of Physical Education, Graduate School, Dongguk University, Seoul 04620, Republic of Korea; 2 Machine Learning Systems Lab., College of Sports Science, Sungkyunkwan University, Suwon 16419, Republic of Korea

**Keywords:** human activity recognition, attention, pattern recognition, sequence classification, wearable device, explainable artificial intelligence

## Abstract

The squat is a multi-joint exercise widely used for everyday at-home fitness. Focusing on the fine-grained classification of squat motions, we propose a smartwatch-based wearable system that can recognize subtle motion differences. For data collection, 52 participants were asked to perform one correct squat and five incorrect squats with three different arm postures (straight arm, crossed arm, and hands on waist). We utilized deep neural network-based models and adopted a conventional machine learning method (random forest) as a baseline. Experimental results revealed that the bidirectional GRU/LSTMs with an attention mechanism and the arm posture of hands on waist achieved the best test accuracy (F1-score) of 0.854 (0.856). High-dimensional embeddings in the latent space learned by attention-based models exhibit more clustered distributions than those by other DNN models, indicating that attention-based models learned features from the complex multivariate time-series motion signals more efficiently. To understand the underlying decision-making process of the machine-learning system, we analyzed the result of attention-based RNN models. The bidirectional GRU/LSTMs show a consistent pattern of attention for defined squat classes, but these models weigh the attention to the different kinematic events of the squat motion (e.g., descending and ascending). However, there was no significant difference found in classification performance.

## 1. Introduction

Human activity recognition (HAR) aims to automatically analyze and understand the signals collected from activities occurring in the real world through various sensors [1]. HAR has demonstrated its effectiveness in many application domains where human movement is the primary target, such as healthcare and rehabilitation [2,3]. Most studies on HAR primarily focus on coarse-grained classification, which classifies motion types with distinct differences between activities, such as walking, running, and sitting. In case of evaluating the *quality* of a certain exercise, a fine-grained HAR is necessary (e.g., good or bad posture) [4,5]. Since fine-grained motion classification aims to distinguish similar motions [6,7,8], the system normally requires machine learning algorithms that can learn subtle differences among similar motion signals.

The bodyweight (BW) squat is a multi-joint exercise widely used for various health related workouts, which has the advantage that it can be performed virtually anywhere without special equipment. However, since there exists a risk of injury depending on how it is performed, maintaining proper postures while squatting is especially important [9]. For example, if squats are repetitively performed with incorrect postures, stress may accumulate on the knee joints, and eventually injuries can occur, such as patellofemoral pain syndrome [10]. Several previous studies have focused on identifying inappropriate postures utilizing a variety of sensor systems [11,12,13,14]. 

For instance, O’Reilly et al. proposed a system to identify seven different squat motions, including knee valgus, heels off, and excessive lean, using a backpropagation neural network (BP-NN) classifier based on a single IMU attached to the fifth lumbar vertebrae [12]. They reported a classification accuracy of 56.55%. In a subsequent follow-up study [14], a different type of wearable system in which the IMU is attached onto the right shank was proposed for recognizing the six predefined squat motions. They reported an enhanced classification accuracy of 73.1% when a random forest algorithm was adopted as a classifier. Recently, Lee et al. [13] proposed a wearable system that can classify six different squat motions based on measurements from an IMU attached to the outside of the right thigh. They achieved a reasonable accuracy of 80.9% by leveraging the learning capabilities of the proposed CNN-LSTM architecture. Although fine-grained classifications using the motion signals from a single embedded sensor were attempted for differentiating squat styles, it is worth noting that exercising while attaching a dedicated sensor on specific body parts (e.g., the trunk and lower extremities) is not feasible as it may raise usability issues, especially when the application is targeted for everyday at-home fitness.

Smartwatches equipped with inertial sensors are highly versatile devices in HAR because they are easy to wear and exhibit high potential for ubiquitous computing [15,16]. Unlike other types of smart devices, such as smartphones, smartwatches exhibit low variability between measurements because the device mount position is normally limited (i.e., non-dominant hand) and the device is in a stable and fixed contact to the body part virtually all the time. One important issue to consider is whether the measured signal delivers sufficient information regarding the quality of exercise. In recent studies, inertial motion information captured by smartwatch can be used for fine-grained recognition of a variety of human activities ranging from daily activities [17,18] to walking and running movements [8,16,19,20]. 

Meanwhile, deep learning is heavily involved in many decision-making processes in various fields. However, in general, the inference results of algorithms are not normally designed to provide human-comprehensible explanations; as such, interpreting the results from deep learning models has long been a missing component. Recently, the attention mechanism has begun to play significant roles in securing the explainability of machine learning algorithms [16,19,21]. The attention-based model was originally proposed to learn the alignment between input and output sequences [22], such as the alignment between visual features in an image and its text description in image caption generation tasks [23], and the alignment between English words and French words in machine translation tasks [24,25]. In case of a sequence classification task where there are only input sequences, the model learns the most relevant parts of the input sequence given a target value. Many recent studies have utilized this alignment as an explainable element of artificial neural network when the underlying decision-making process needs to be explained [8,19,26,27]. For example, a recent study proposed an intelligent wearable system for recognizing human activities using multiple sensors [19]. The system could determine the timesteps of data that contributed the most during the decision-making process with the attention mechanism. Similar to this, we previously proposed and validated activity recognition systems for estimating walking-related activities [8,16] based on an attention-based neural network.

In this paper, based on a hypothesis that the proposed squat recognition process can be formulated as a supervised learning problem, we propose a smartwatch-based wearable system that can recognize fine-grained squat motions with deep neural networks along with a recent attention mechanism. To validate the feasibility of the proposed approach, we conducted an experiment to collect a squat dataset from 52 university undergraduate and graduate students (age: 27.01 ± 5.1; 31 men, 21 women). We trained and tested the dataset in an end-to-end fashion using a set of supervised machine learning algorithms, including 1D-CNN and gated RNNs, such as GRU and LSTM. Focusing on the observation that not all motions contribute equally to the process of determining whether the action was performed well, we also incorporated an attention-mechanism into the proposed squat evaluation system. By leveraging the explainability of the attention mechanism, we further analyze the motion classification process based on attention vectors. The primary contributions of this paper are as follows:

1. We formulated a machine learning problem for recognizing fine-grained differences in a specific at-home fitness activity (squat) in a supervised learning fashion.
2. We incorporated an attention mechanism for identifying the relative contributions of the motion sequence data during the decision-making process by the machine-learning system.
3. We visualized and analyzed the machine-generated attention vectors during the inference phase.

## 2. Materials and Methods

### 2.1. Measurement Setting and Data Collection

#### 2.1.1. System for Data Collection

**Figure 1:**
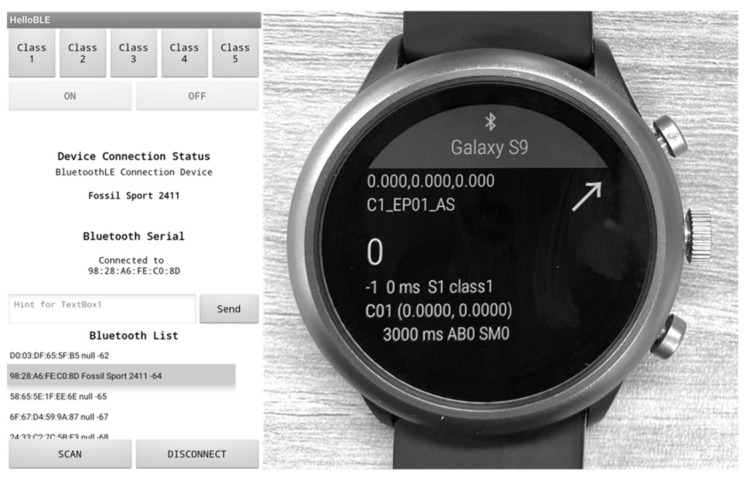
Custom host (smartphone) application and wearable (smartwatch) application developed for this study.

**Figure 2:**
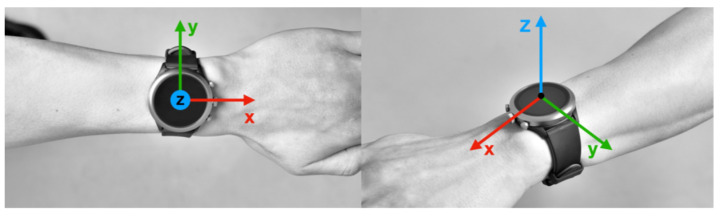
The smartwatch used in this study with its axis displayed.

For data collection, we employed a commercially available smartwatch, Fossil FTW4019 (Fossil Inc., Richardson, TX, USA), which is equipped with an IMU (including an accelerometer and a gyroscope) and runs on the Google WearOS platform. We developed a custom wearable application for the smartwatch to capture motion properly and a custom host application to remotely control the smartwatch and monitor its status over Bluetooth, as Figure 1 shows. The sampling rate was set to 50 Hz during the experiment, which is the fastest option for the device and sufficient for capturing the characteristics of human activity [28]. During the data collection phase, all participants wore the watch on their non-dominant wrist (all participants reported this as left), as Figure 2 shows.

#### 2.1.2. Definition of Squat Class

The squat is a multi-joint exercise widely conducted as a health-related workout, which has the advantage of being able to be performed virtually anywhere without special equipment. A standard squat can be quantitatively evaluated by appropriate flexion of the hips, extension of the spine, and direction of the knees according to the guidelines of the National Strength and Conditioning Association [29]. Insufficient hip flexion, excessive lumbar flexion, and valgus of the knee are typical incorrect squat motions. Insufficient hip flexion is characterized by a lack of hip range of motion while squatting. Although this limited range of motion does not directly cause injuries, it can be an indicator of functional deterioration and the risk of injury in the knee and ankle. Excessive lumbar flexion, or bending the upper body too much while performing the squat, may increase the likelihood of low back injury [30]. Thus, maintaining the correct back alignment during squats is important to ensure spine stability. Knee valgus refers to the knee collapsing inward as the hip flexes while performing the squat. The knee valgus motion is considered a major risk factor for anterior cruciate ligament rupture and patellofemoral pain syndrome [31]. 

**Figure 3:**
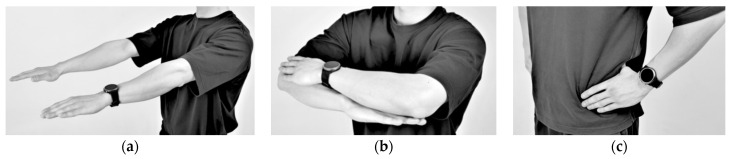
Arm postures: (**a**) straight arms, (**b**) crossed arms, and (**c**) hands on waist.

Since recognition of one’s own movements during exercise is normally challenging if there are no means of external supervision [32,33], such issues appear frequently in squats. Therefore, it is important to monitor whether the exercise is being conducted in the right posture. In this context, we formulated a supervised machine learning problem based on six different squat styles, including a correct posture, three different incorrect squat postures, and two different combinations of incorrect postures, as Table 1 shows. While collecting the data, we also constrained the arm postures to three typically used ones: straight arm (SA), crossed arm (CA), and hands on waist (HW), as Figure 3 shows. Thus, each participant was asked to conduct the predefined 18 squat motions (6 squat classes × 3 arm positions) per session.

#### 2.1.3. Participants

A total of 52 participants, (31 men and 21 women) without any experiences of musculoskeletal injuries in the past one year, were selected for data collection. The average age, height, and weight of the participants were 27.0 (±5.1) years, 171.5 (±8.1) cm, and 70.3 (±12.5) kg, respectively. Thirty people (18 men and 12 women) participated as the experienced practician group who performed weight training, including squat movements, for more than a year, and 22 people (13 men and 9 women) were considered novice participants. All of them reported themselves as right-handed. All participants were informed of the experimental procedure, which was approved by the Institutional Review Board of Dongguk University (DUIRB-202109-14).

#### 2.1.4. Procedure

Participants in the experiment were instructed by fitness experts in both the correct and incorrect squat postures, and a warm-up period was assigned prior to the main experiment. During the main experiment, we employed a metronome to provide participants with temporal cues of the descending and ascending phases of the motion (six-eight time, 120 bpm, dotted quarter note). Each participant performed ten sessions, composed of six motion classes with three different arm postures in random order. Thus, a total of 7800 squats (1,547,200 data points) were collected from 52 participants (10 sessions × 3 arm postures × 6 classes × 52 participants). Participants were allowed to take a rest whenever required.

### 2.2. Data Preprocessing

Table 2 summarizes the time and number of data points in collected data for each of the six classes (C1 through C6). The total data collection time was 386.8 min. All the data were normalized by applying standard scaling for further processing.

### 2.3. Data Segmentation

**Figure 4:**
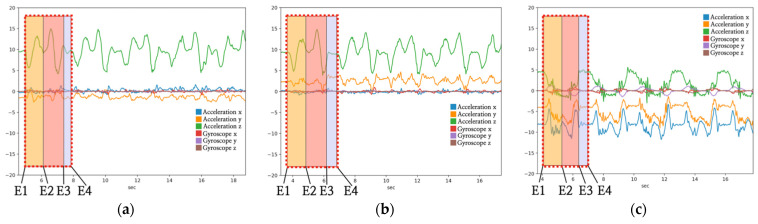
Examples of manual segmentation with respect to arm postures: (**a**) straight arms (SA), (**b**) crossed arms (CA), and (**c**) hands on waist (HW). Segmented data are highlighted in the dotted black rectangle. Motions highlighted in orange (E1–E2), red (E2–E3), and blue (E3–E4) rectangles are the descending, ascending, and standing phase, respectively. Here, E1 denotes the point at which the change in the acceleration begins with descent, E2 indicates the point at which the acceleration decreases after attaining the maximum descent point, E3 represents the point at which the ascent is completed after the maximum descent, and E4 denotes the point immediately before the start of the subsequent squat.

Although it is known that a single squat normally takes 2 to 4 s, the time required to perform a single squat varies depending on the participant’s ability, squat styles, and external conditions. Thus, segmenting data with a fixed-sized window may not be suitable for our case in that it may ultimately lead to low classification performance [34]. In this study, we segmented the motion data by manually inspecting the recorded video and time-series motion data, such that a segmented data contains one full cycle of the squat. The average interval of segmentation window was 2.95 ± 0.14 s. When manually segmenting the windows (i.e., deciding the beginning and end of a squat), human inspectors mainly examined sensor values and only watched video data when required. Figure 4 shows examples of segmentation highlighted in dotted red rectangles. The variable length of segmented data is handled by padding zeros on the right during the training phase.

### 2.4. Classification Algorithm

#### 2.4.1. Feature-Based Machine Learning: Random Forest

We adopted the random forest (RF) algorithm as a baseline classifier as it demonstrates robust and accurate results in many machine-learning problems [35,36,37]. It is categorized as an ensemble machine learning method that combines multiple classifiers. An RF comprises *n* trees that produce *n* classification results because each decision tree comprising the RF is a separate classifier. The final output is determined through a majority vote on the classification result through bagging. Typically, RF demonstrates high and robust classification performance compared to other types of feature-based machine-learning algorithms. Table 3 shows features used for this study, which are selected according to the feature significance test [38].

#### 2.4.2. Deep Learning-Based Models

We adopted recent deep-learning algorithms, including one-dimensional convolutional neural networks (CNNs) and gated recurrent neural networks (RNNs), such as long short-term memory unit (LSTM) and gated recurrent unit (GRU), for learning the captured time-series data in a supervised learning fashion.

- One-Dimensional (1D) CNN

**Figure 5:**
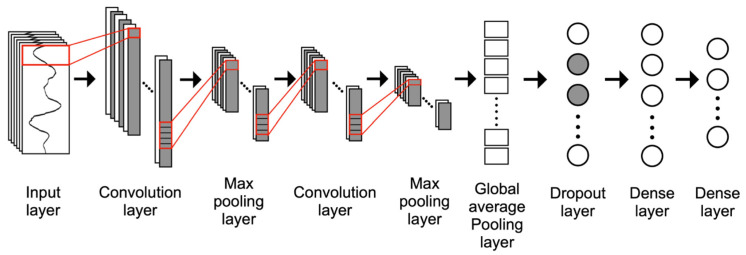
Architecture of the one-dimensional convolutional neural network (1D-CNN).

CNN is a particular type of artificial neural network designed to adaptively extract spatial hierarchies of features [40]. It efficiently extracts local features by restricting the receptive fields to be local [41]. During the last few years, it has demonstrated its effectiveness in many application fields that deal with images [42], spectral data [37], 3D volumes [43], and sequential data [44,45,46], to name a few. The one-dimensional version of CNN, called 1D-CNN, is also widely studied to learn time-series data in a convolutional manner [16,41,44,46]. The 1D-CNN used in this study comprises two convolution layers and a max pooling layer to extract local features and reduce dimensions, respectively, as shown in Figure 5, and a global average pooling layer that converts channels into 1D vectors to be input to subsequent layers. The kernel size was set to 3, and the stride length was set to 1.

- LSTM and gated recurrent unit (GRU)

**Figure 6:**
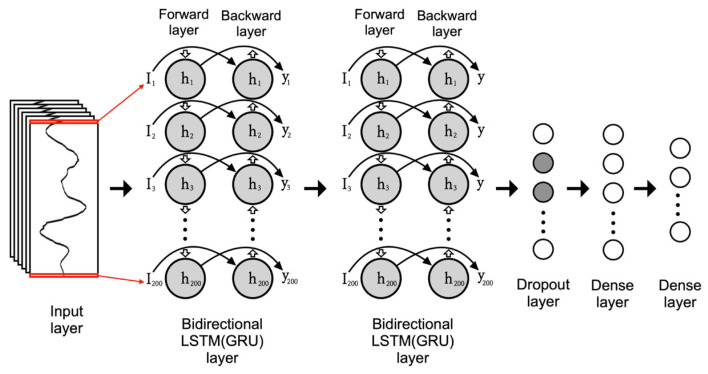
Architecture of bidirectional long short-term memory (LSTM) and gated recurrent unit (GRU).

Although the standard RNN is designed to learn for discovering intricate structure in sequential data [27], it suffers from the vanishing and exploding gradient problem. To mitigate this issue, LSTM was proposed by incorporating the memory-cell and gate units into the RNN structure to encapsulate information about long-term dependencies [47]. GRU, which is structurally similar to LSTM, is a model developed to update parameters to capture dependencies of different time scales adaptively [48,49]. Here, gates can be seen as a way to selectively let information through. It has outperformed the standard RNN algorithm in many sequence transduction tasks [48,50,51] and classification tasks [8,52,53,54]. Additionally, we utilized constructed models with LSTM and GRU with bidirectional wrappers, which allowed input data to be processed along positive and negative time directions, as Figure 6 shows.

- Attention Mechanism

**Figure 7:**
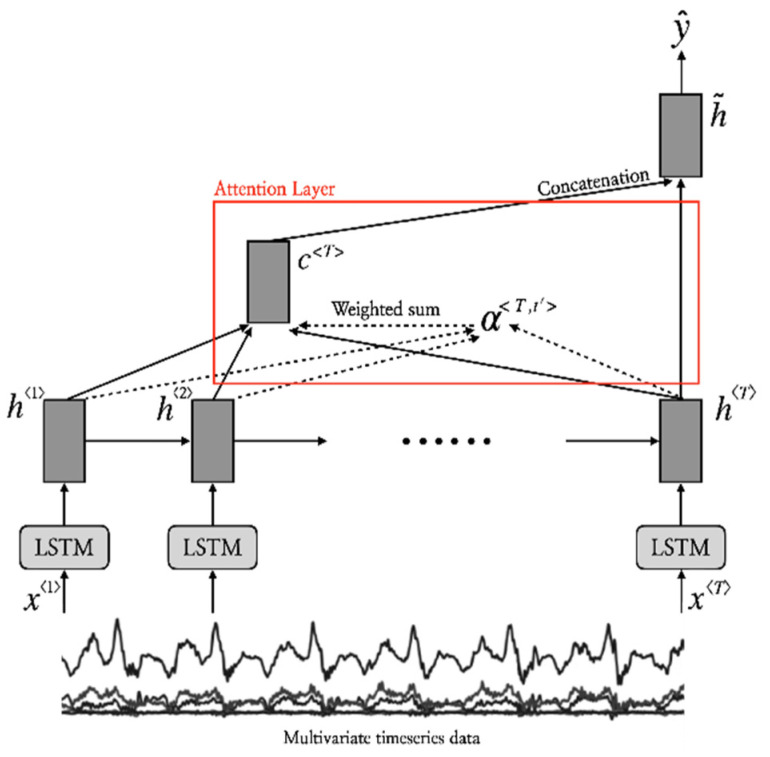
Multiplicative attention-based LSTM/GRU [22] for the proposed activity classification process.

The attention mechanism is designed to allow artificial neural networks to focus on specific parts of the input data, similar to human attention, and it has arguably become one of the most important building blocks in recent artificial neural networks [55,56]. In this study, we adopted a multiplicative attention mechanism, which reduces encoder/decoder states to an attention score via a simple matrix multiplication [22]. For the classification task, the mechanism calculates the relevance score between the last hidden state at T and a linear transformation of the hidden state of LSTM/GRU at $t^{\prime }$ as:

(1) $$\alpha^{\langle T,t^{\prime }\rangle }=\mathrm{softmax}\left(\mathrm{score}{\left(h^{\langle T\;\rangle },\;h^{\langle t^{\prime }\rangle }\right)}_{t^{\prime }=1}^{T}\right)\;=\frac{\exp\left(h^{\langle T\rangle }W_{a}h^{\langle t^{\prime }\rangle }{}^{⊤}\right)}{\sum_{t^{\prime }=1}^{T}\exp\left(h^{\langle T\;\rangle }h^{\langle t^{\prime }\rangle }{}^{⊤}\right)}$$

where $\mathrm{score}\left(\cdot \right)$ is a bilinear function that compares the two hidden states of LSTM/GRU, and $W_{a}$ is the trainable weight matrix of attention. The attention score, $\alpha^{\langle T,t^{\prime }\rangle }$, describes the amount of attention that target value $\hat{y}$ should pay to the input feature at time $t^{\prime }$ (i.e., $h^{\langle t^{\prime }\rangle }$). In this paper, we utilize the attention score $\alpha^{\langle T,t^{\prime }\rangle }$ as an explainable element of the deep learning system. Figure 7 shows details of the attention mechanism used in this study.

## 3. Results

### 3.1. Classification Results

We employed F1-score, recall, precision, accuracy, and a confusion matrix to evaluate the classification performance of the proposed method. Here, accuracy is the ratio of samples that are classified correctly in the entire sample.

(2) $$Accuracy=\frac{True\;positive+True\;negative}{True\;positive+True\;negative+False\;positive+False\;negative}$$

The F1-score indicates the harmonic mean of recall and precision:

(3) $$F_{1}=\frac{2\times Precision\times Recall}{Precision+Recall}$$

Precision denotes the ratio of samples that are true positive among the samples predicted to be true and false positive, whereas recall is the ratio of samples that are predicted to be true positive among the samples that are true positive and false negative.

The classification results of each classifier for six squat classes were analyzed in terms of test and train accuracies and F1-scores with respect to the arm postures.

#### 3.1.1. Baseline Results from a Random Forest

Table 4 summarizes the results from the RF classifier. Overall, the manual segmentation condition exhibited higher classification performance. Additionally, it is worth noting that the performance with the test dataset is significantly degraded compared to that with the training dataset, meaning that RF has a robustness issue when generalizing the trained model to unseen data in our case. 

#### 3.1.2. Results Using a Deep Neural Network Models

Table 5 summarizes the classification results with respect to the arm postures; the HW condition demonstrates the best classification results across all the models. 1D-CNN exhibited the lowest classification performance, except in the CA arm posture. Overall, results obtained from the deep neural networks were higher than those from the baseline classifier, RF.

**Figure 8:**
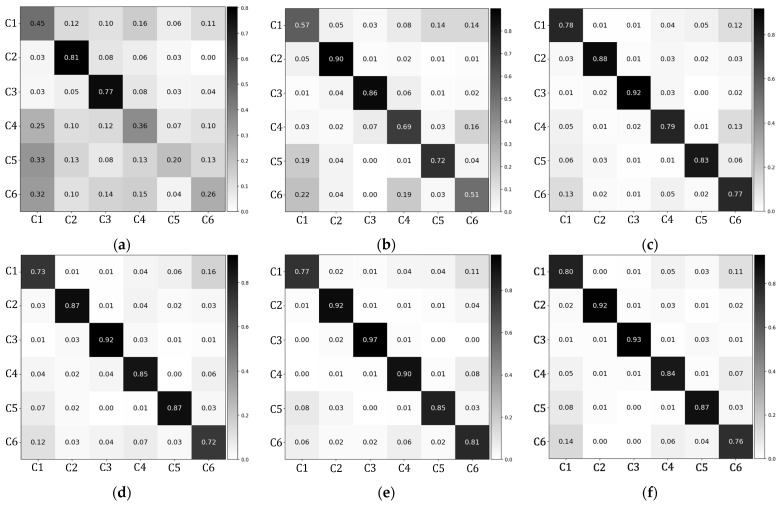
Confusion matrices of HW condition with respect to the neural network models employed: (**a**) random forest; (**b**) 1D-CNN; (**c**) bidirectional LSTM; (**d**) bidirectional GRU; (**e**) BiLSTM with attention; (**f**) BiGRU with attention.

**Figure 9:**
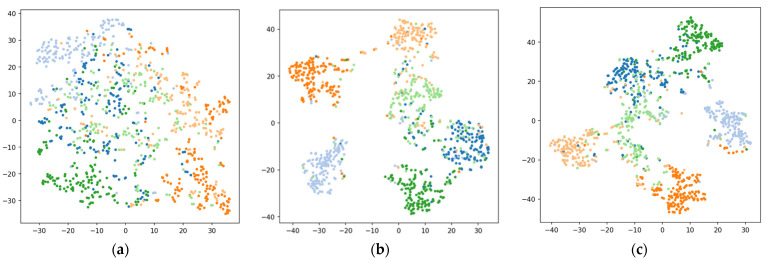

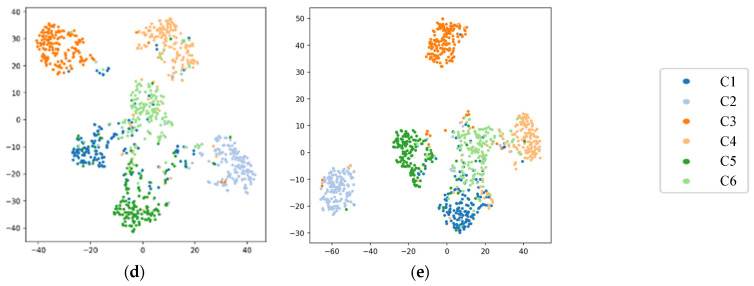
t-SNE visualization of high-dimensional (D = 64) learned features by deep-learning models in the HW condition: (**a**) 1D-CNN; (**b**) bidirectional LSTM; (**c**) bidirectional GRU; (**d**) BiLSTM with attention; (**e**) BiGRU with attention. Motion classes are indicated by colors. Each two-dimensional point represents a segmented motion of T = 200 that is projected from the 64-dimensional feature space. Embeddings produced by attention-based models exhibit more clustered distributions than those by other DNN models, indicating that attention-based models learned features from the complex multivariate time-series (MTS) motion signals more efficiently.

Figure 8 illustrates the confusion matrices for each deep neural network model. We adopted t-distributed stochastic neighborhood embedding (t-SNE) for visualizing high-dimensional feature spaces learned in each model [57]. Figure 9 shows the two-dimensional embeddings projected from the 64-dimensional representations of the last hidden layer using the t-SNE algorithm.

## 4. Discussion

### 4.1. State of the Art in Squat Exercise Recognition with Smartwatch

Experimental results obtained indicate that DNN models exhibited higher classification performance than the feature-based baseline model (i.e., RF), as reported in [8,16,44,58]. This may be because hand-crafted features are not sufficient for the fine-grained recognition task of squat activities. The 1D-CNN model achieved the lowest classification performance among the DNN models employed in our study, which is not consistent with results reported in a recent work [8] in which the 1D-CNN demonstrated higher classification performances over other types of DNN models. 

Our attention-based models demonstrated better classification performances than those used in the previous studies that employed a single sensor for the squat activity recognition [12,13,14]. The attention-based models lead to better data clustering than those without an attention mechanism, as shown in Figure 9. The results of the clustering performance using normalized mutual information (NMI) also show that models with the attention mechanism exhibited higher NMI values compared to those from models without the attention mechanism, as Table 6 shows. On the other hand, the clusters in the embedding space learned by the 1D-CNN model are not as clearly clustered in the latent space as those learned by other DNN models. This phenomenon is also supported by the NMI score obtained from the 1D-CNN model shown in Table 6. Note that this is significantly lower than the NMI scores from other DNN models. 

The classification performance is also affected by the arm posture. Since the HW condition provides stable hand supports while squatting, the results from the HW condition exhibited the best classification results. Additionally, as the measuring device is mounted onto a fixed body part (i.e., the wrist of the non-dominant hand), there may exist limitations in measuring all the subtle changes in motion, especially those originating from body parts on the other side of the body (e.g., the right-knee). In fact, it was found that there are systematic confusions made between the squat with correct posture (C1) and the squat with both-knee valgus (C6), as shown in Figure 8 and Figure 9.

### 4.2. Explainable and Trustworthy AI Coaching System Based on Attentional Neural Mechanisms

**Figure 10:**
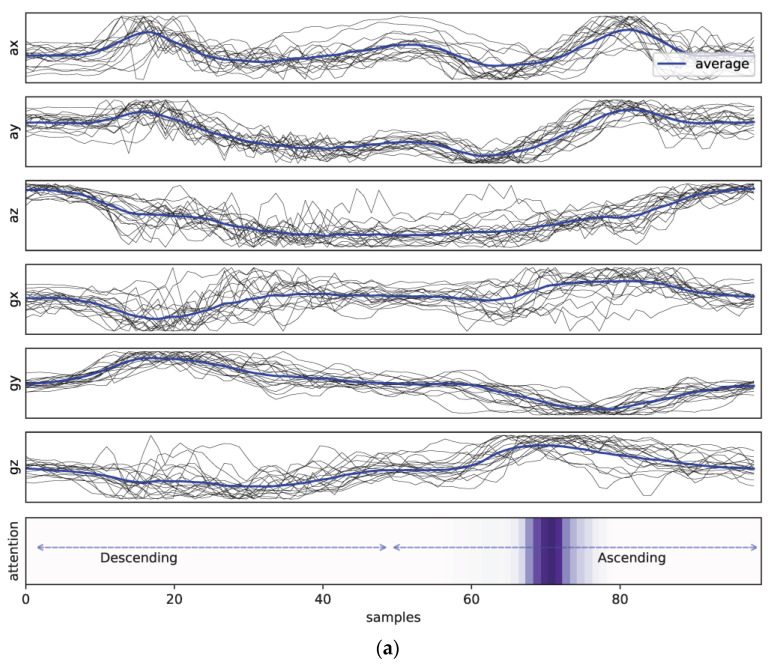

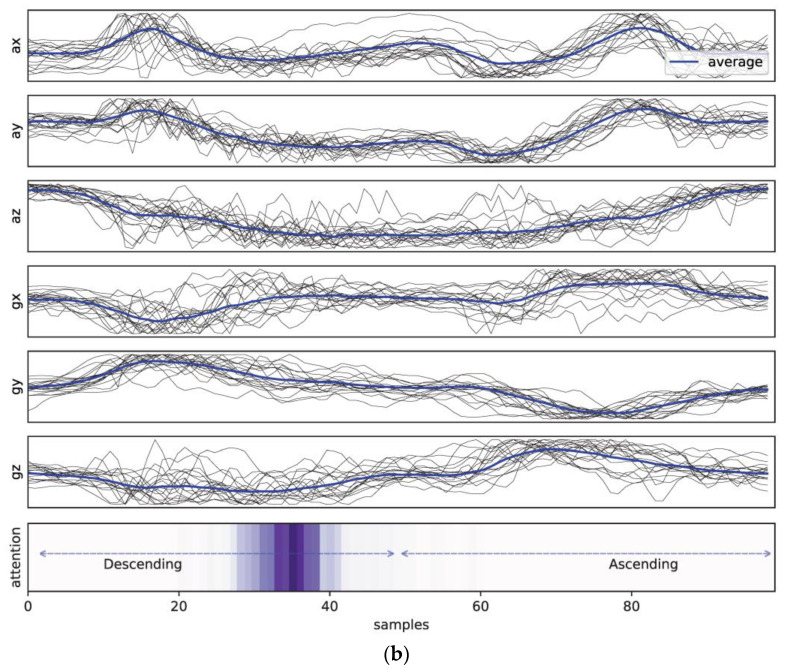
Example of MTS input signals (grey line) from same squat activities (normal squat) with temporally aligned attention vectors highlighted (averaged): (**a**) BiLSTM with attention; (**b**) BiGRU with attention. Averaged MTS input signals are highlighted by blue solid lines. The darker the highlighted bar, the more attention it received and overlapped from the model (other classes are described in Figure A1 and Figure A2). Note that the BiGRU model systematically tends to focus on the descending phase while the BiLSTM model on the ascending phase of the squat motion during the decision-making process.

A deep neural network can extract the required features to discover intricate structures in low-level sensor readings [59]. However, it is unclear how the model comes to a specific decision in the classification process. Thus, learning an interpretable representation has become an essential task in many machine learning problems [60]. Similarly, we tried to identify the parts of the input signals that most contributed to the prediction results (i.e., good squats or types of bad squats, etc.) by incorporating an attention mechanism into our classification models. Figure 10 illustrates examples of the visualization of averaged attention vector from BiLSTM and BiGRU models and the averaged raw sensor signals in correct squat class (C1). The length of input sequences is normalized to 101 timesteps. The darker the highlighted area, the more attention received from the model during the inference phase. It was found that the parts of the input signals that contribute most during the decision-making process depend on the types of base recurrent units: the BiGRU model tends to focus on the descending phase when making a prediction, whereas the BiLSTM model on the ascending phase of the squat motion. In our study, the differences between the two models were found to be consistent across all classes of squat motions (see Appendix A). Despite such differences, the classification performances of these two models were similar (F1-scores of BiLSTM and BiGRU are 0.871 and 0.856, respectively).

### 4.3. Limitations and Further Work

Several limitations were identified in this study. Firstly, in terms of classification performance, the trained model in this study may have a generalization issue, as only samples with limited distributions were collected during the data acquisition phase. In fact, we faced challenges when collecting motions with bad postures (C2 through C6) compared to those with correct postures (C1). Since the acquisition of all types of incorrect motions is infeasible, we plan to extend our research to improve the robustness of the model by formulating an unsupervised task (e.g., anomaly detection) as future work.

Secondly, we did not validate the dataset through computer vision. Since we collected data using a wrist-worn sensor, we did not record the kinematic feature that could have been observed at joints during squat motions. To validate machine learning algorithms and establish the gold standard, we plan to validate datasets using computer vision in our future work.

Thirdly, although we investigated the attention vectors to identify parts of the input signals that most contribute during the decision-making process, we have not examined how machine-generated attentions are correlated with the opinions from human experts. We believe that this process is essential for fine-grained exercise analysis of at-home fitness activities, as in other domains [55], to provide users with more informed decisions.

## 5. Conclusions

In this paper, we proposed a smartwatch-based wearable system that can recognize the subtle motion differences produced while squatting. To that end, we formulated a supervised learning problem in which five different incorrect squat motions, each of which may increase the likelihood of injuries, and a one correct motion are to be recognized. The captured signals from the smartwatch on the wrist were trained and evaluated in the proposed task with recent deep neural network models, including 1-D CNN and gated RNNs (e.g., LSTM and GRU) optionally with an attention mechanism (i.e., multiplicative attention mechanism). Experimental results revealed that the BiGRU/LSTMs with an attention mechanism achieved a reasonable test accuracy (F1-score) of 0.854 (0.856), which is higher than those from the other machine learning approaches employed in this study. We further analyzed the attention vectors produced during the inference phase to understand the relative contributions of the MTS signals in the classification process. It is noticeable that the BiGRU model systematically tends to focus on the descending phase while the BiLSTM model focuses on the ascending phase of the squat motion during the decision-making process. In our future work, we plan to extend our research by formulating an unsupervised task (e.g., anomaly detection) to improve the robustness of the proposed approach.

## Figures and Tables

**Table 1:** Squat motion defined in this study.

Class	Description	Figure	Class	Description	Figure
C1	Normal	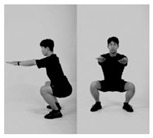	C4	Left-knee valgus	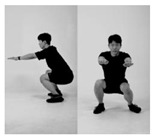
C2	Insufficient depth	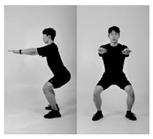	C5	Right-knee valgus	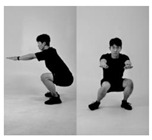
C3	Insufficient depth with posterior tilting and knee valgus	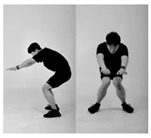	C6	Both-knee valgus	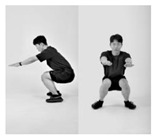

**Table 2:** Durations of the collected squat data.

Arm Posture	Class	Min (Hour)	Data Points (Train)
Straight arm (SA)	C1	25.8 (0.43)	103,200 (72,000)
	C2	25.9 (0.43)	103,500 (72,000)
	C3	25.95 (0.43)	103,800 (72,000)
	C4	25.9 (0.43)	103,600 (72,000)
	C5	25.3 (0.42)	101,200 (68,000)
	C6	25.95 (0.43)	103,800 (72,000)
Crossed arm (CA)	C1	25.95 (0.43)	103,800 (72,000)
	C2	25.9 (0.43)	103,600 (72,000)
	C3	25.1 (0.42)	100,400 (68,000)
	C4	25.6 (0.43)	102,400 (68,000)
	C5	25.85 (0.43)	103,400 (72,000)
	C6	25.7 (0.43)	102,800 (68,000)
Hands on waist (HW)	C1	25.9 (0.43)	103,600 (72,000)
	C2	25.95 (0.43)	103,800 (72,000)
	C3	25.95 (0.43)	103,800 (72,000)
	C4	25.95 (0.43)	103,800 (72,000)
	C5	25.8 (0.43)	103,200 (72,000)
	C6	25.8 (0.43)	103,200 (72,000)
Total		464.25 (7.72)	1,856,900 (1,280,000)

**Table 3:** Examples of features selected based on the feature significance test. Adapted with permission from [38,39]. 2016, Maximilian Christ and Blue Yonder GmbH.

Feature Name	Descriptions
fft_coefficient	Fourier coefficients of the one-dimensional discrete Fourier transform for real input by fast Fourier transform algorithm
change_quantiles	Average, absolute value of consecutive changes of the time series inside the corridor
abs_energy	Absolute energy of the time series, which is the sum over the squared values
variance	Variance of time series
standard_deviation	Standard deviation of time series
absolute_sum_of_changes	Sum over the absolute value of consecutive changes
Root_mean_square	Root mean square (rms) of the time series
mean_abs_change	Average over first differences
ratio_value_number_to_time_series_length	Factor, which is 1 if all values in the time series occur only once, and below one if this is not the case
linear_trend	A linear least-squares regression for the values of the time series versus the sequence from 0 to length of the time series minus one

**Table 4:** Accuracy and F1-score of random forest.

Arm Posture		
Straight Arm (SA)	Test/Train accuracy	0.609/0.73
	Test/Train F1-score	0.591/0.718
Crossed Arm (CA)	Test/Train accuracy	0.619/0.725
	Test/Train F1-score	0.62/0.726
Hands on Waist (HW)	Test/Train accuracy	0.533/0.703
	Test/Train F1-score	0.512/0.696

**Table 5:** Accuracy and F1-score when deep neural networks were used.

Arm Posture		1D-CNN	Bidirectional LSTM	Bidirectional GRU	BiLSTM with Attention	BiGRU with Attention
Straight Arm (SA)	Test/Train accuracy	0.513/0.628	0.61/1.0	0.571/1.0	0.663/1.0	0.635/1.0
	Test/Train F1-score	0.507/0.624	0.61/1.0	0.569/1.0	0.663/1.0	0.633/1.0
Crossed Arm (CA)	Test/Train accuracy	0.597/0.707	0.641/1.0	0.653/1.0	0.663/1.0	0.568/1.0
	Test/Train F1-score	0.586/0.702	0.64/1.0	0.651/1.0	0.663/1.0	0.565/1.0
Hands on Waist (HW)	Test/Train accuracy	0.711/0.781	0.828/1.0	0.829/1.0	0.871/1.0	0.854/1.0
	Test/Train F1-score	0.71/0.78	0.83/1.0	0.829/1.0	0.871/1.0	0.856/1.0

**Table 6:** Normalized mutual information (NMI) on test dataset.

Title 1	Arm Postures		
	SA	CA	HW
Random forest	0.339	0.314	0.243
1D-CNN	0.301	0.355	0.533
Bidirectional LSTM	0.363	0.391	0.703
Bidirectional GRU	0.323	0.400	0.663
BiLSTM with attention	0.471	0.503	0.739
BiGRU with attention	0.396	0.416	0.732

**Figure A1:**
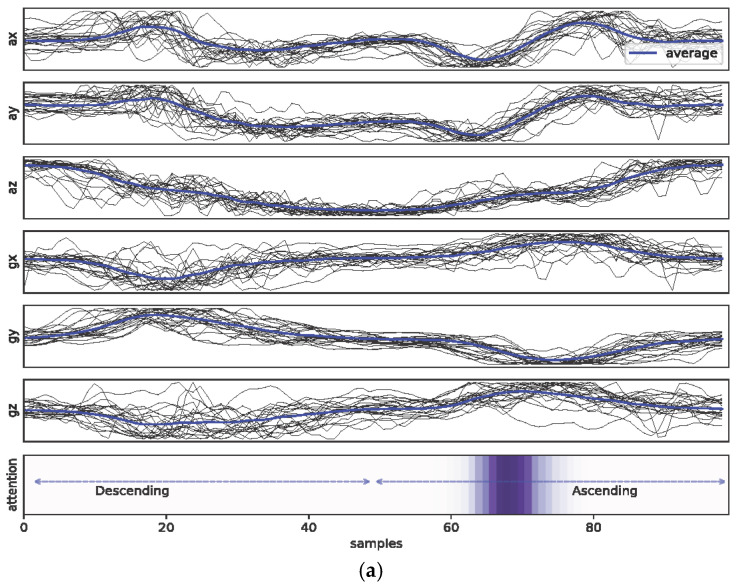

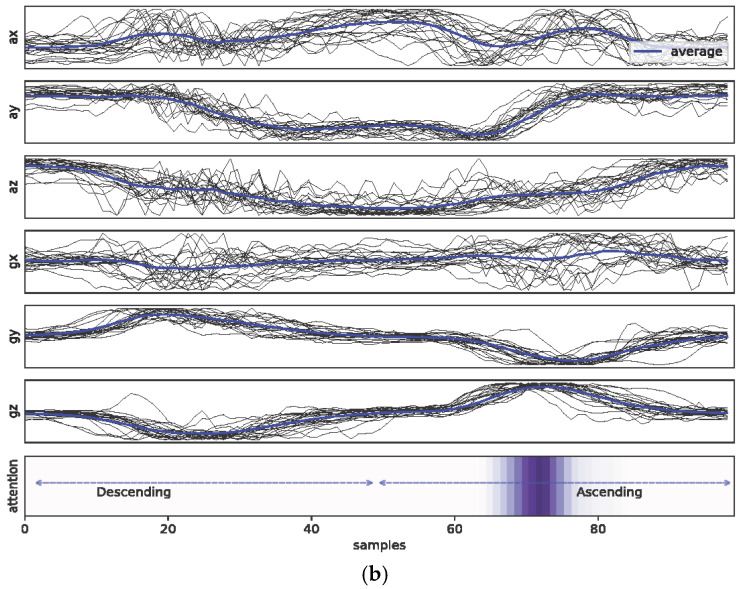

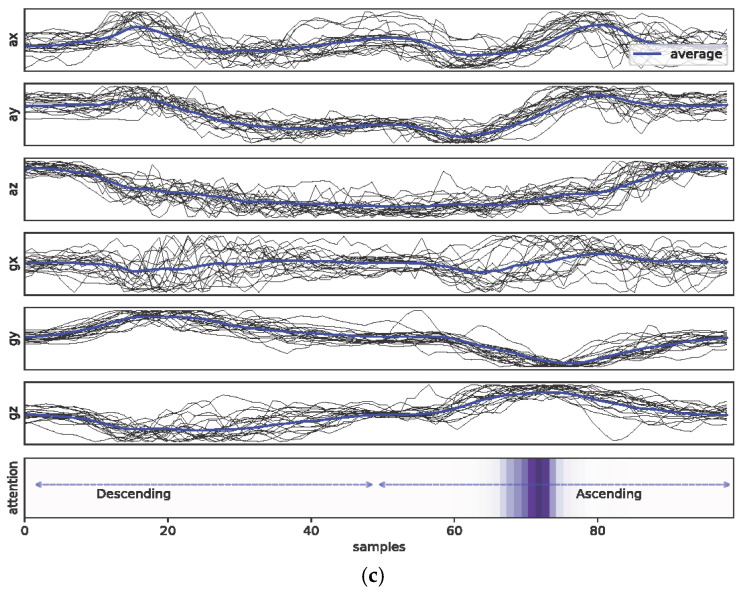

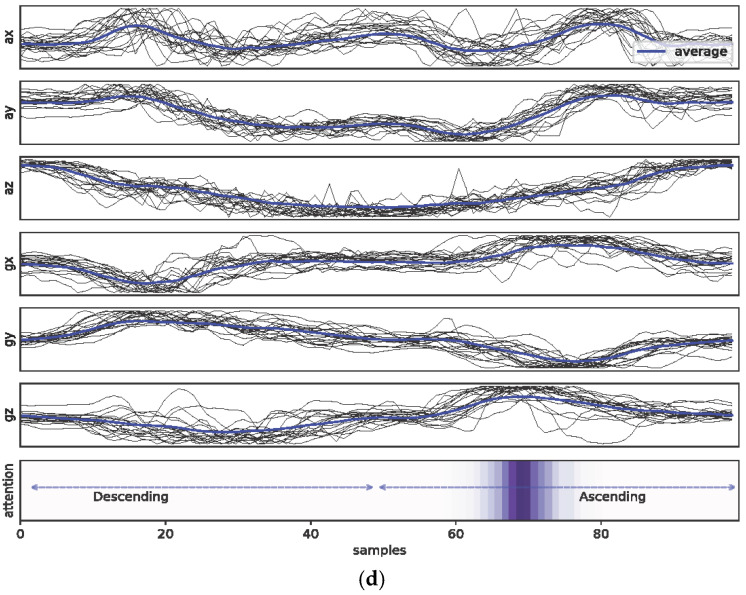

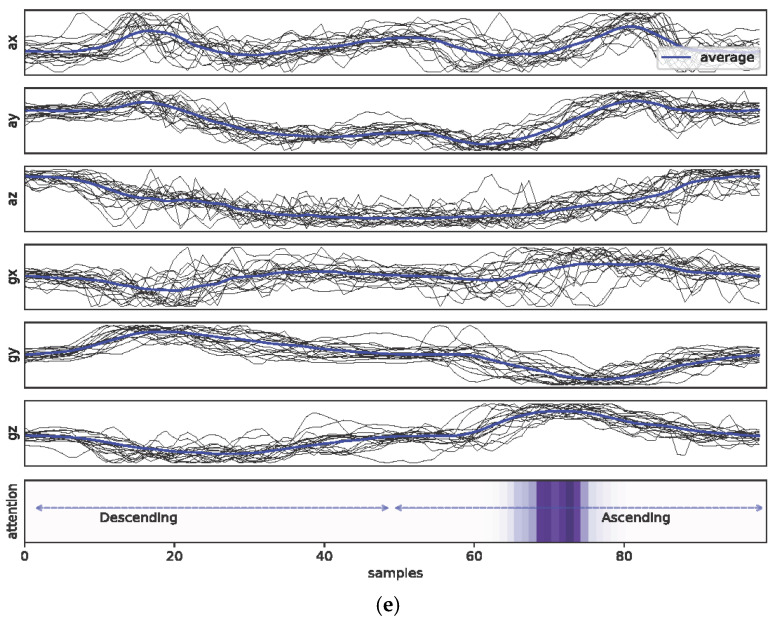
Examples of MTS input signals (grey line) with temporally aligned attention vectors highlighted (averaged) based on BiLSTM with attention: (**a**) C2; (**b**) C3; (**c**) C4; (**d**) C5; (**e**) C6.

**Figure A2:**
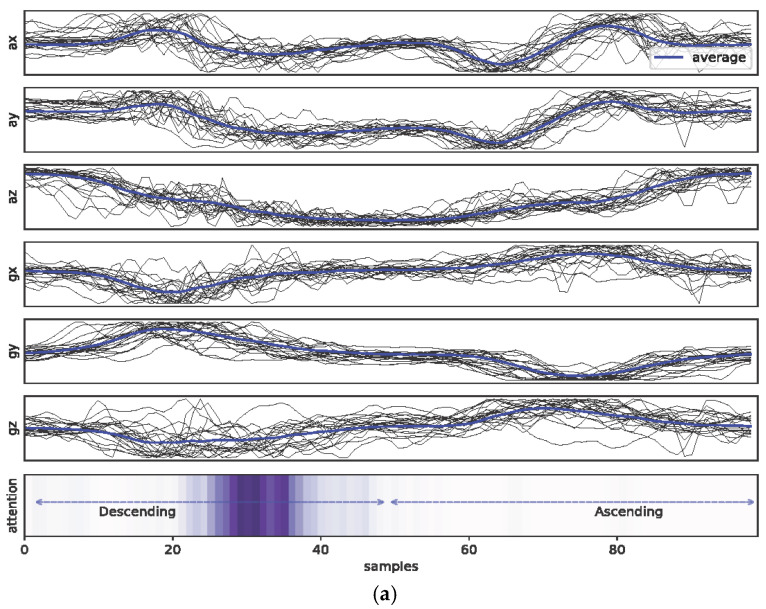

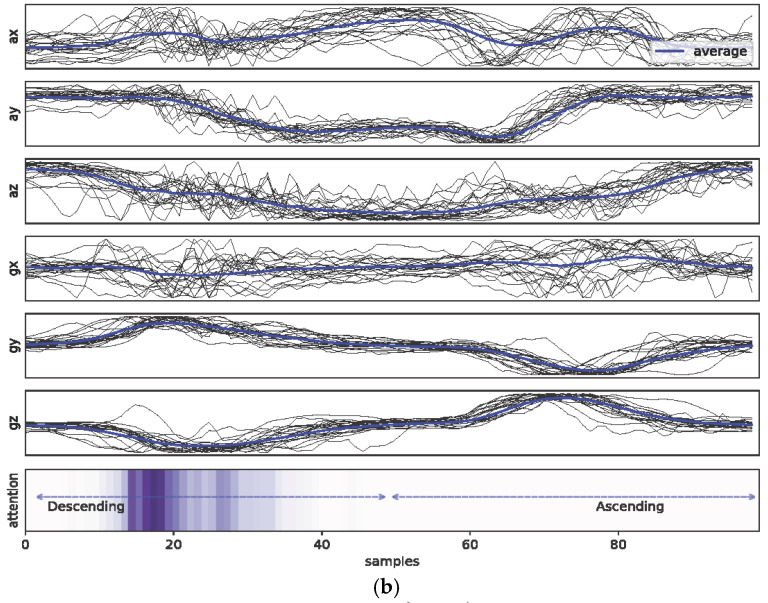

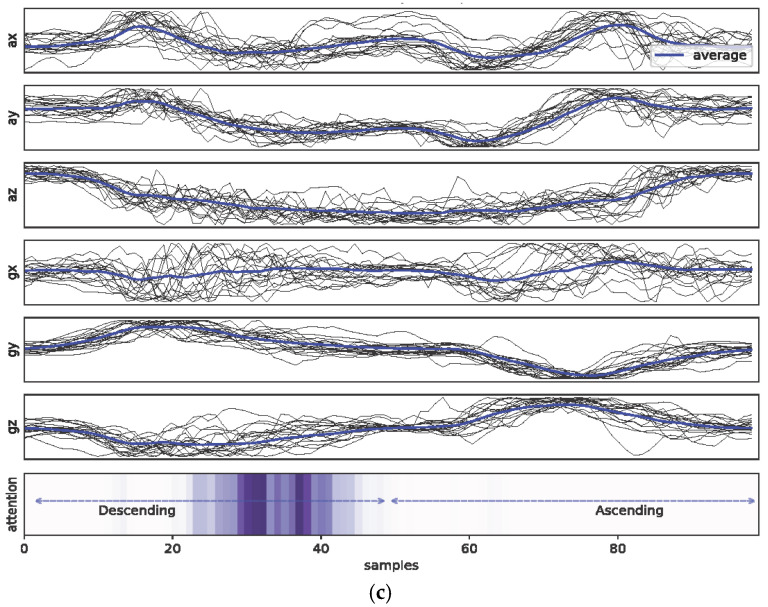

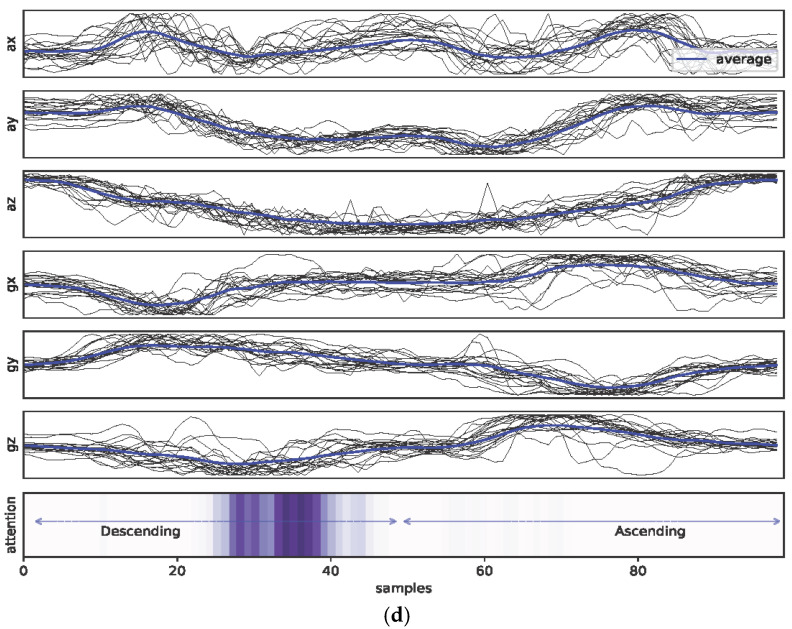

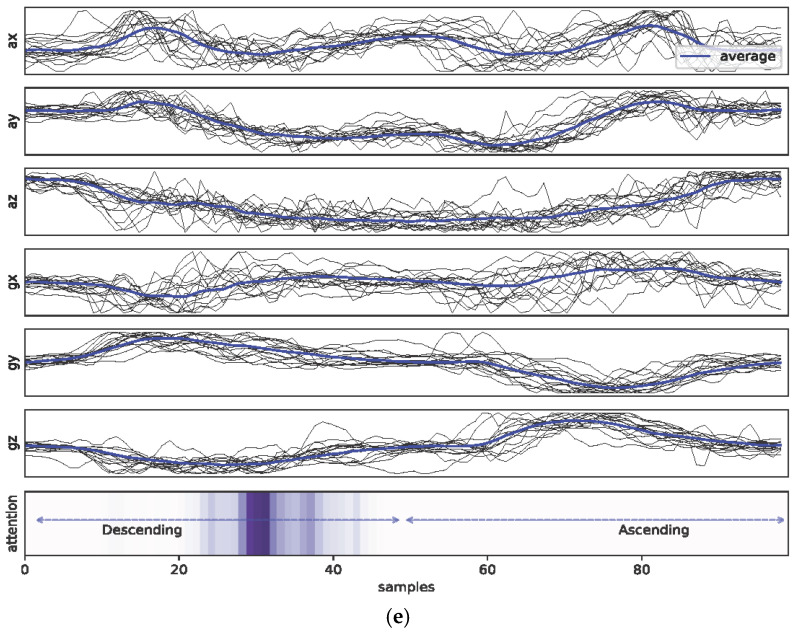
Examples of MTS input signals (grey line) with temporally aligned attention vectors highlighted (averaged) based on BiGRU with attention: (**a**) C2; (**b**) C3; (**c**) C4; (**d**) C5; (**e**) C6.

## Data Availability

The raw data supporting the conclusions of this article will be made available by the authors, without undue reservation.
